# Purtscher-like retinopathy presented a honeycomb-like pattern in optical coherence topography angiography

**DOI:** 10.1186/s12886-019-1233-8

**Published:** 2019-11-21

**Authors:** Bing Li, Donghui Li, Youxin Chen

**Affiliations:** Department of Ophthalmology, Peking Union Medical College Hospital, Key Lab of Ocular Fundus Diseases, Chinese Academy of Medical Sciences & Peking Union Medical College, Beijing, 100730 People’s Republic of China

**Keywords:** Purtscher-like retinopathy, Fundus fluorescein angiography, Optical coherence topography angiography; optical coherence topography

## Abstract

**Background:**

To report a case of Purtscher-like retinopathy (PUR) and the optical coherence tomography (OCT) and OCT angiography (OCT-A) findings before and after treatment.

**Case presentation:**

A 65-year-old male presented with acute onset of vision loss for 2 weeks. Fundus examination revealed cotton-wool spots, retinal haemorrhage, and Purtscher flecken spread around the optic disc in the right eye. He was diagnosed with Purtscher-like retinopathy because he lacked any traumatic medical history. OCT presented some band-like hyperreflective lesions at the inner nuclear layer, which are indicative of paracentral acute middle maculopathy (PAMM). OCT-A revealed apparent reduction in blood flow signal at the deep retina and choriocapillaris layers with a honeycomb-like hypointense signal pattern. After 3 months of follow-up, OCT revealed resolution of retinal oedema, but PAMM lesions remained visible. Based on OCT-A, the honeycomb-like pattern turned into a homogeneous reduction in blood flow with small patches of hypointense signal areas in the choriocapillaris.

**Conclusion:**

This case presented a new OCT-A sign in PUR with a honeycomb-like hypointense signal at the choriocapillaris layer, indicating the involvement and ischaemia of the choroid during the pathological process.

## Background

Purtscher’s retinopathy was first described in 1910 by Otmar Purtscher in a middle-aged man who fell off a tree and suffered cranial trauma [1]. When non-traumatic aetiologies are present, the correct definition is Purtscher-like retinopathy (PUR). The exact mechanism of Purtscher’s retinopathy/PUR remains unknown. The most accepted theory for its pathogenesis is attributed to an embolic phenomenon resulting in occlusion of the precapillary arterioles [2]. Here, we report a case of PUR with some notable signs in optical coherence tomography (OCT) and OCT angiography (OCT-A), which may provide more information about the pathological features.

## Case presentation

**Fig. 1:**
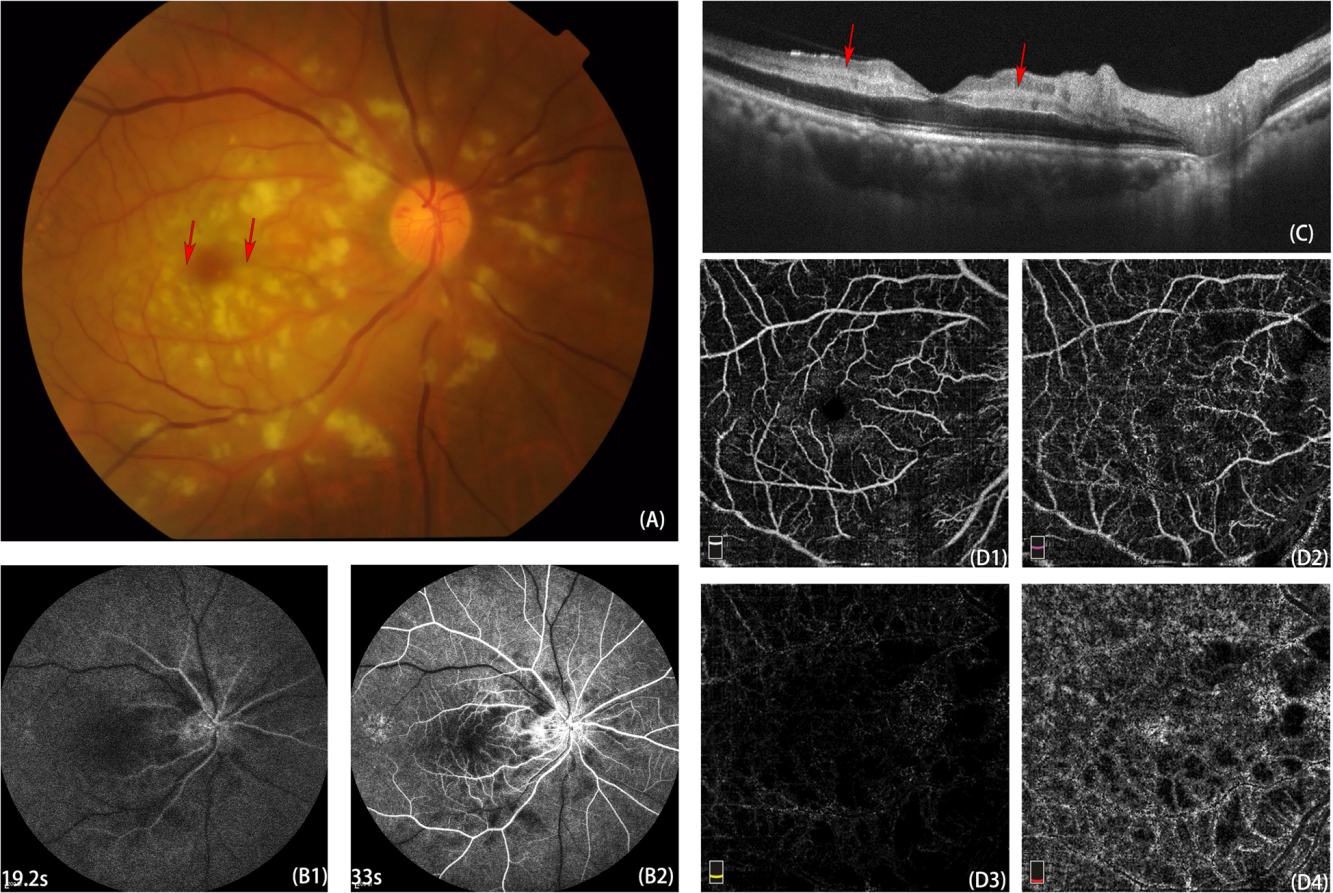
Multimodal imaging of the affected right eye. Fundus photography showed extensive spreading of cotton-wool spots confined to the peripapillary and posterior pole with slight retinal haemorrhage, as well as Purtscher flecken around the macular fovea in the right eye. **a**. FA presented delayed arteriovenous circulation time (**B1**, **B2**). OCT B-scan of the fovea noted retinal oedema and PAMM-like lesion at INL (red arrows)(**c**). OCT-A of the fovea (6 × 6 mm) indicates the condition of blood supplement in superficial retina (**D1**), outer retina (**D2**), deep retina (**D3**) and choriocapillaris (**D4**). The blood flow reduced apparently in both the inner and deep retinal vascular plexuses and a honeycomb-like hypointense signal pattern at the choriocapillaris layer

A 65-year-old male presented to our clinical center with the chief complaint of acute onset of vision loss in the right eye for 2 weeks without a clear cause before the onset. He reported neither trauma nor a special systemic medical history. Ophthalmic inspections showed that the best-corrected visual acuity (BCVA) was hand movements in the right eye and 20/20 in the left eye. The affected right eye presented a positive result of relative afferent pupillary defect (RAPD) test. Other signs of anterior segment and intraocular pressure were unremarkable. Dilated fundus examination revealed extensive spreading of cotton-wool spots confined to the peripapillary and posterior pole with slight retinal haemorrhage, as well as Purtscher flecken around the macular fovea in the right eye. The contralateral eye was almost normal except for mild arteriosclerosis. Fluorescence angiography (FA) indicated a slight delayed arteriovenous circulation time (14 s) and mottled hypofluorescence corresponding to cotton-wool spots and Purtscher flecken. OCT presented retinal thickening and oedema, especially at the inner layer. In addition, OCT revealed lesions of the hyperreflective band at the inner nuclear layer (INL) and on either side of the fovea corresponding to perifoveal wedge-shaped white-gray lesions, which is similar to paracentral acute middle maculopathy (PAMM). OCT-A revealed reduced blood flow in both the inner and deep retinal vascular plexuses and a honeycomb-like hypointense signal pattern at the choriocapillaris layer. (Fig. 1).

**Fig. 2:**
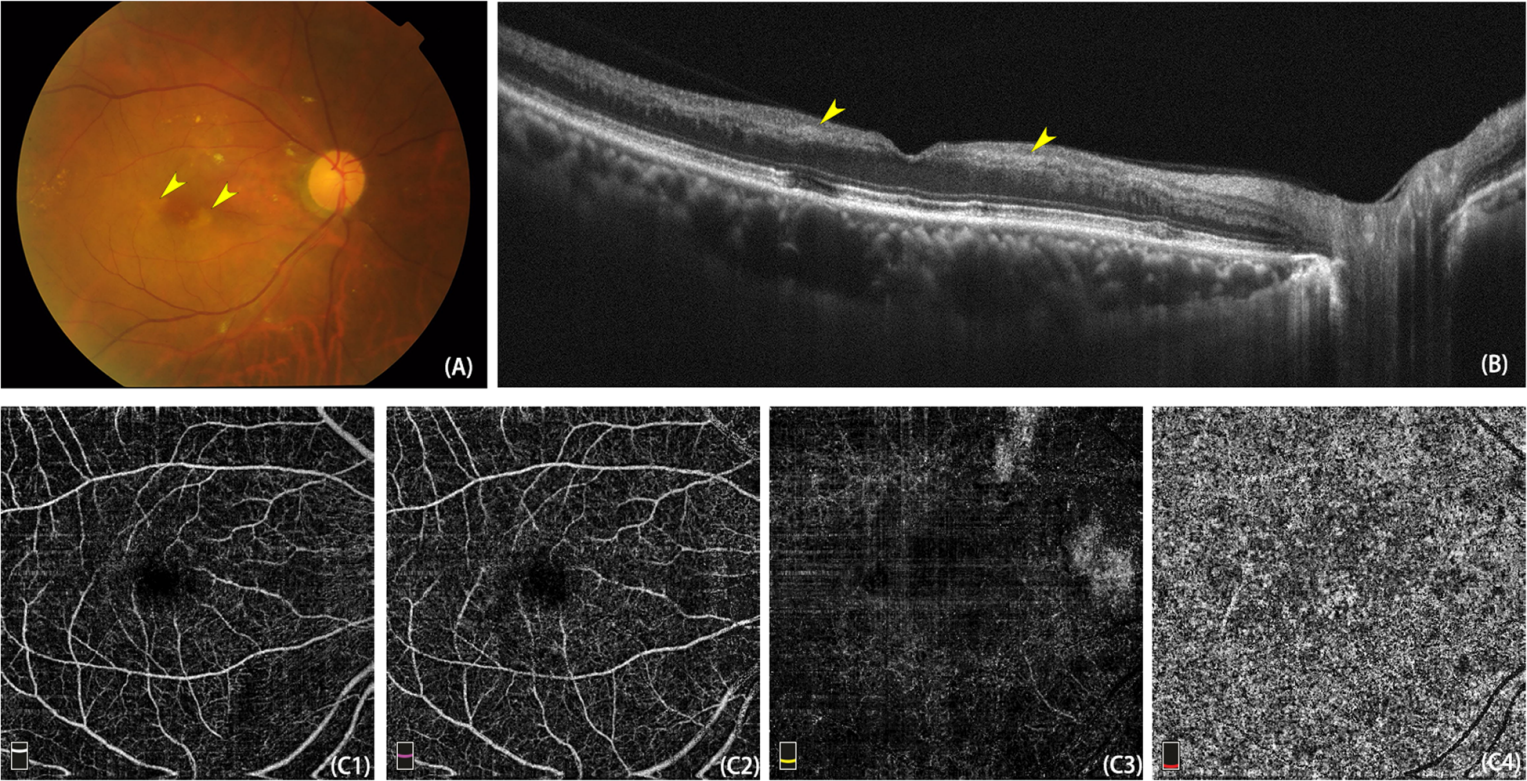
After 3 months of follow-up, the retinal lesions were mostly absolved (**A**), OCT noted PAMM lesions corresponding to perifovea wedge-shaped white-grey lesions (**B**, yellow arrowhead). OCT-A showed blood flow of different layers in retina and choroid: superficial retina (**C1**), outer retina (**C2**), deep retina (**C3**) and choriocapillaris (**C4**). OCT-A presented a hypoperfusion condition in both inner and outer retina with some small patches of hypointense signal areas

He was diagnosed with PUR due to the lack of trauma or other specific systemic diseases. We ordered laboratory testing to exclude common aetiologies, such as systemic lupus erythaematosus (SLE) and thrombotic thrombocytopenic purpura (TTP). The results, including antinuclear antibodies (ANA), antineutrophil cytoplasmic antibody (ANCA), erythrocyte sedimentation rate (ESR), C-reactive protein (CRP) and routine blood examination, were all within normal limits. He was also referred to the internal medical department for further investigation. Only obsolete lacunar infarctions were detected after a thorough review of the whole body. *Alprostadil* (Pfizer, ATTN: CAVERJECT®) 10 U intravenous injection (Q.D. for 10 days) was prescribed as vascular dilation therapy to improve retinal blood flow and prevent further damage. After 3 months of rehabilitation, his visual acuity recovered to 20/400. The retinal haemorrhage, cotton wood spots and Purtscher flecken were mostly resolved. OCT indicated remission of retinal oedema, but the PAMM lesions (the hyperreflective band at the INL) remained visible. OCT-A revealed that the honeycomb-like pattern in the choriocapillaris had turned into a homogeneous reduction in blood flow with some small patches of hypointense signal areas. (Fig. 2).

## Discussion and conclusions

According to a recent systemic review of PUR, the most frequent aetiologies are trauma and acute pancreatitis [3]. A previous report presented a patient who developed PUR after a myocardial infarction with a concomitant transient ischaemic attack [4]. Although the exact mechanism of PUR remains unknown, currently, the most accepted theory for its pathogenesis is an embolic phenomenon resulting in occlusion of the precapillary arterioles [2]. Air, fat, platelets, fibrin, leukocyte aggregates and exogenous particles are all potential emboli. In this case, the presence of lacunar infarction hinted that fundus disorders share the same aetiology as the retinal vasculature has embryologic origins similar to those of the cerebral vasculature. Hypertensive retinopathy is an important differential diagnosis. However, his blood pressure was well controlled at that time and was unilaterally involved without apparent papilloedema; thus, this diagnosis seems less likely.

The diagnosis of PUR is mostly clinical depending on specific medical histories, including sudden vision loss after trauma or other special systemic diseases and typical signs, including retinal haemorrhage, cotton-wool spots and Purtscher flecken. Multimodal-image inspections, including OCT, OCT-A and FA, may provide more information for diagnosis and follow-up. Previous researchers have reported common signs of FA findings in PURs, including areas of non-perfusion, retinal ischaemia and slower filling of vessels [2]. OCT-A may reveal extensive non-perfusion in the macular area in both the inner and deep capillary plexuses [5, 6]. OCT mostly indicates retinal oedema at the macula. Recent studies have reported cystoid macular oedema or even subretinal fluid in patients with PUR [7]. PAMM lesions, a special OCT feature of PUR discovered in recent years, present as hyperreflective bands at the INL in OCT, indicating an ischaemic condition at the outer retinal capillary plexus [8].

This patient also presented low perfusion in the retina layer. However, the presentation of low perfusion in the choriocapillaris was characteristic with a notable sign of a honeycomb-like hypointense signal pattern in OCT-A, indicating the ischaemic involvement of the choroid. The anatomical features of the lobular blood supply in the choroid from the short posterior ciliary artery may partly explain this phenomenon since the areas of low perfusion are localized and well defined. The reduction in flow in the choriocapillaris is easily considered a projection artefact of cotton-wool spots and flecken of the superficial layer. However, the low perfusion area is more extensive and shows clearer boundaries in D4 than in D2. The projection artefact would not have such sharp edges for low perfusion areas.

Many patients with PUR can regain their visual acuity to normal levels spontaneously after the aetiology is resolved, while some patients have a poor prognosis despite various treatments. There is still no exact prognostic factor for PUR. In this case, some special circumstances should be taken into consideration to predict visual outcomes. Because the patient delayed consultation for 2 weeks, the exact process of the disease was unknowable, and the best opportunity for intervention might have been lost. Additionally, the presence of PAMM lesions surrounding the fovea indicated a poor prognosis as this sign represented the ischaemia of both the inner and deep retinal capillary plexuses at the fovea [9]. Furthermore, OCT-A showed a honeycomb-like hypointense signal pattern in choriocapillaris that corresponds to anatomic structures of choroidal lobules. This finding provided a sign of choroidal lobular infarction and explained the restricted recoverability of this patient. At present, there is still no consensus on the treatment of PUR. Most viewpoints consider observation and treatment of the underlying aetiology to be the most reasonable therapeutic option without the risk of adverse drug effects. In this case, we prescribed Alprostadil injections, which have been proven to attenuate immunohistochemical and histological repercussions in renal tissue [10]. In addition, we referred him to the internal medicine department for further examination for cerebral infarction and hypertension, which may be the underlying aetiology of PUR.

In conclusion, PURs are reportedly mostly related to trauma or other special systemic conditions. PUR is largely an embolic occlusion disease. This case provides another perspective to detect potential embolic aetiologies, which is especially reasonable for patients in their 60s if no trauma or other specific systemic diseases are reported. FA and OCT-A provide more information for diagnosis and follow-up visits. The presence of PAMM lesions on OCT scans and a honeycomb-like hypointense signal pattern in OCT-A at the choriocapillaris layer may indicate a poor visual prognosis because of the ischaemia in the macular fovea and choroid.

## Data Availability

Some datasets generated and/or analyzed during the current study are not publicly available due to the article word limit, but are available from the corresponding author on reasonable request.

## Abbreviations

ANA: Antinuclear antibodies
ANCA: Antineutrophil cytoplasmic antibody
CRP: C-reactive protein
ESR: Erythrocyte sedimentation rate
FA: Fluorescein angiography
INL: Inner nuclear layer
OCT: Optical coherence topography
OCT-A: Optical coherence topography angiography
PAMM: Paracentral acute middle maculopathy
PUR: Purtscher’s/Purtscher-like retinopathy
SLE: Systemic lupus erythematosus
TTP: Thrombotic thrombocytopenic purpura

## References

[1] Purtscher O (1910). Noch unbekannte befunde nach schadeltrauma. Ber Dtsch Ophthalmol Ges.

[2] Agrawal A, McKibbin MA (2006). Purtscher's and Purtscher-like retinopathies: a review. Surv Ophthalmol.

[3] Miguel AI, Henriques F, Azevedo LF, Loureiro AJ, Maberley DA (2013). Systematic review of Purtscher's and Purtscher-like retinopathies. Eye (London, England).

[4] Ang L, Chang BCM (2017). Purtscher-like retinopathy - a rare complication of acute myocardial infarction and a review of the literature. Saudi J Ophthalmol.

[5] Hamoudi H, Nielsen MK, Sorensen TL (2018). Optical coherence tomography angiography of Purtscher retinopathy after severe traffic accident in 16-year-old boy. Case Rep Ophthalmol Med.

[6] Xiao W, He L, Mao Y, Yang H (2018). Multimodal Imaging in Purtscher Retinopathy. Retina (Philadelphia, Pa).

[7] Onaran Z, Akbulut Y, Tursun S, Ogurel T, Gokcinar N, Alpcan A (2019). Purtscher-like retinopathy associated with synthetic cannabinoid (Bonzai) use. Turk J Ophthalmol.

[8] Rahimy E, Kuehlewein L, Sadda SR, Sarraf D (2015). Paracentral Acute Middle Maculopathy: What We Knew Then and What We Know Now. Retina (Philadelphia, Pa).

[9] Nakashima H, Iwama Y, Tanioka K, Emi K. Paracentral acute middle Maculopathy following Vitrectomy for proliferative diabetic retinopathy: incidence, risk factors, and clinical characteristics. Ophthalmology. 2018. 10.1016/j.ophtha.2018.07.006 30126649

[10] Soares BL, Freitas MA, Montero EF, Pitta GB, Miranda F (2014). Alprostadil attenuates inflammatory aspects and leucocytes adhesion on renal ischemia and reperfusion injury in rats. Acta Cir Bras.

